# External Communication Barriers among Elderly Deaf and Hard of Hearing People in China during the COVID-19 Pandemic Emergency Isolation: A Qualitative Study

**DOI:** 10.3390/ijerph182111519

**Published:** 2021-11-02

**Authors:** Di Xu, Chu Yan, Ziqing Zhao, Jiaying Weng, Shiwen Ma

**Affiliations:** 1 School of Journalism and Information Communication, Huazhong University of Science and Technology, Wuhan 430074, China; yanchu@hust.edu.cn (C.Y.); zhaoziqing@hust.edu.cn (Z.Z.); mashiwen@hust.edu.cn (S.M.); 2 School of Medicine and Health Management, Tongji Medical College, Huazhong University of Science and Technology, Wuhan 430030, China; wengjiaying@hust.edu.cn

**Keywords:** elderly deaf and hard of hearing (DHH) people, COVID-19, emergency isolation, external communication barriers, Wuhan

## Abstract

The COVID-19 pandemic poses a great risk to older people with hearing impairment, who face a higher threshold of external communication after the implementation of the emergency isolation policy. As part of a study on the optimization of external communication among the deaf and hard of hearing (DHH) population in central China, this study employed a qualitative research method based on in-depth interviews to explore the needs and difficulties faced by the older DHH group in external communication during public health emergencies in Wuhan, China, in the context of the COVID-19 pandemic. The results showed that older DHH people had weak reception of critical information about the epidemic, and had suboptimal access to medical care during emergency quarantine, which increased interpersonal communication barriers to this group. The current findings highlight the urgent need for targeted strengthening of the original emergency communication and coordination mechanisms in public health emergencies, and for improving policy inclusiveness for older DHH individuals during the COVID-19 pandemic and emergencies alike.

## 1. Introduction

COVID-19 has been a global outbreak [1], and on 30 January 2020, the World Health Organization declared the COVID-19 outbreak a global health emergency of international concern. To control and prevent further spread of the outbreak, China activated the highest level of emergency response and implemented severe emergency quarantine measures in compliance with the Law on Prevention and Treatment of Infectious Diseases (LPTID) [2].

People with disabilities are one of the groups at greater risk of infection, protection pressure and difficulty of treatment in major infectious disease outbreaks, and are also often overlooked in disasters [3]. In Wuhan, China, the initial epicenter of the epidemic, 13,000 people with hearing loss were registered [4]. As of 11 February 2020, the Wuhan Deaf Association (a non-profit organization voluntarily formed by deaf people and some social organizations, enterprises, institutions and individuals working with deaf people in Wuhan) counted seven confirmed and nine suspected covid cases, and two additional cases of illness and death in Wuhan [5]. According to the annual tracking and monitoring data of the Hubei Disabled Persons Federation, the older disabled population aged 60 and above in Wuhan accounts for 50.27% of the total disabled population in the city, which includes 29.9% of the older deaf and hard of hearing (DHH) population [6].

Previous studies have confirmed that age is a risk factor for serious outcomes from COVID-19. According to statistics, more than 90% of COVID-19-associated deaths in the UK occur in people over 60 years of age [7]. In addition to the deteriorating health status associated with physical aging, the prevalence of hearing impairment increases with age [8]. Recent studies showed that older DHH people may suffer exponentially more physical and mental effects during the COVID-19 pandemic when both hearing impairment and old age are present [9], some of whom often encounter information, communication and healthcare barriers [10,11]. These populations could be more vulnerable than hearing older adults [12]. As a matter of fact, older individuals with hearing loss have a higher chance of having chronic conditions such as stroke, diabetes, hypertension and asthma [13], with a high prevalence of some chronic conditions, this population is more likely to visit hospitals or emergency departments during a COVID pandemic, where has a higher likelihood of being exposed to COVID-19 infection [9,14]. As a consequence, when elderly DHH people who have both hearing impairment and old age may suffer exponentially more physical and psychiatric impact, and be at risk of heightened social isolation and loneliness [15,16], as well as encounter more unusual adaptive barriers [17], during a COVID pandemic. Therefore, the smoothness of external communication in this group becomes particularly important.

Globally, the importance of external communication is a key area of concern for social activists and people with disabilities alike [18]. Because people with hearing loss are born with communication shortcomings, the hearing barrier poses many difficulties for this group, and the biggest challenge they face in the COVID-19 outbreak often comes from poor external communication [19,20]. During the implementation of the extraordinary social isolation policy, the temporary closure of various channels and institutions undoubtedly aggravated the external communication predicament of the DHH group [21]. The importance of doctor–patient communication increases accordingly during the COVID-19 pandemic, and many DHH people will actively seek medical help during this period. Therefore, this study included doctor–patient communication, which is often involved in emergency situations of public health emergencies, as a dimension of external communication to be examined. The term “external communication” in this study includes not only the epidemic information acquisition and interpersonal communication behaviors of older DHH people in normal situations, but also the doctor–patient communication behaviors of this group in emergency situations. Studies from Korea and the Netherlands have shown that strong isolation measures and social distancing have a significant negative impact on the lifestyles of older adults [22,23]. If older DHH individuals are unable to successfully communicate externally during the social isolation of a COVID pandemic, they may not only be unable to access the latest information about the pandemic and deepen their adaptive difficulties in daily life, but also be unable to secure the same medical entitlements as the general population [4,24].

The principal aim of this study is to understand the implications of COVID-19 outbreak on older DHH people in Wuhan, China, during emergency quarantine in the context of the COVID-19 epidemic. The objectives for this study included analyzing their external communication needs with respect to their living and medical conditions during social isolation, and identifying the issues causing by communication barriers that need to be urgently addressed in this group during public health emergencies. Moreover, this study focuses on exploring the difficulties and obstacles of external communication among the DHH population, intending to ameliorating the situation by drawing lessons from the experience in Wuhan, and making suggestions on directions and countermeasures to optimize policy inclusion for older DHH people during the COVID-19 pandemic.

## 2. Materials and Methods

### 2.1. Study Design

This study is a qualitative study based on in-depth interviews [25] which aims to understand more comprehensively the real situation of communication among older people with hearing impairment during the COVID-19 pandemic, and to investigate in depth the information access and demand expression as well as the communication barriers of medical consultation and treatment of this group during the emergency blockade period.

### 2.2. Research Team

Members of the research team have over two years of experience volunteering in local sign language, and the second, third and fourth authors are able to communicate fluently with deaf people through local natural sign language. The research team also has long-standing and stable research collaborations with three volunteer teams and public interest organizations for the DHH people. During the social isolation period, members joined a team of emergency assistance volunteers formed on an ad hoc basis to provide interpreters and other public services for deaf people in the local natural sign language (hereafter referred to as “local sign language”, as opposed to official Chinese sign language which is also known as CSL) used in Wuhan. At the time of this study, an attending physician from the department of otolaryngology, Maternal and Child Hospital of Hubei Province, Tongji Medical College, Huazhong University of Science and Technology, provided ongoing advice to the research team on medical specialties.

### 2.3. Respondent Recruitment

Respondents in this study were DHH residents of Wuhan, China, aged 60 years or older, from eight urban areas, including East–West Lake, Hannan, Hanyang, Jiangan, Jianghan, Qiaokou, Qingshan, and Wuchang districts. In accordance with the regulations of the “People’s Republic of China Disability Card” [26], the respondents were persons with hearing disability of Disability Category Code 2 (defined by China Disabled Persons’ Federation, different code numbers represent different disability categories, Code 2 = hearing disability; while Code 1 = visual disability, Code 3 = speech disability, etc.), and all of them were members of the China Disabled Persons’ Population Database and held a disability card issued by the China Disabled Persons’ Federation.

The respondents voluntarily agreed to participate in this study and clearly understood the purpose of the study. The initial interview list was provided by the Wuhan Deaf Association, and the research team conducted a purposive sampling to obtain a preliminary interview list, and then conducted a snowball sampling to expand the list. The final number of interviewees was 13. Among them, 5 were female and 8 were male. Most of them had high school education or below (*n* = 7), and lived in the central city (*n* = 11). The categories according to hearing impairment were deaf (*n* = 7) and hard-of-hearing (*n* = 6). In China, *The* *Lexicon of Common* *Expressions in Chinese National Sign Language* was published on 9 March 2018 and has been officially implemented since 1 July 2018 [27]. In recent years, China has continued to increase the level of popularity of Chinese Sign Language (CSL) and has been gradually implemented nationwide. However, due to age and customary factors, all respondents in this study still used local natural sign language, and none were proficient in CSL. More than half of the respondents experienced illnesses during the social isolation (*n* = 7), of which six are suffering chronic diseases such as cardiovascular diseases, diabetes, tumors, and renal failure. One was hospitalized for acute appendicitis, a sudden acute illness, and one was cured by self-medication at home (Table 1).

### 2.4. In-Depth Interviews

Considering the specificity of the respondents, the research team used peer-to-peer offline in-depth interviews and strictly adhered to the semi-structured interview guidelines to conduct personal conversational interviews [25]. The interviews began with an unstructured open-ended question (“How did the COVID-19 pandemic affect your communication during the social isolation period?”), and then moved to a more specific question about whether communication had changed compared to the non-pandemic period. With the consent of the respondents, the research team recorded detailed personal basic information related to the respondents before the interview and use an audio and video recording equipment to record the whole process of the interview. A total of eight face-to-face interviews were conducted in October–November 2020 in the communities where the respondents lived, with each interview lasting between 1.5 and 3 h. The interview sites were mainly provided by the local community councils, which are grassroots organizations under the jurisdiction of each district federation of persons with disabilities, with dedicated members serving persons with disabilities. The research team ensured that one or two sign language association volunteers were present during each interview to assist the research team with local sign language communication and sign language interpretation of the interview text. All the hard-of-hearing interviewees indicated that they were more familiar with local natural sign language than CSL, and that they could communicate with deaf people in local natural sign language. Two of the six hard-of-hearing respondents wore hearing aids to complete the interview, while the remaining four completed the interview through a combination of written communication and sign language. Some of the interviewees were accompanied to the interview site by friends and relatives, but the accompanying persons would not be included in the formal interviews.

### 2.5. Data Analysis

After all interviews were completed, the research team compiled the interview data into transcripts that were given to the second and third authors of the research team who had more than 2 years of experience volunteering in local sign language, with the third author having a family member who was a teacher at the deaf school. These two authors cross-checked the transcripts with the non-verbal information presented by the interviewees in the original audio and video recordings to ensure accuracy. All the original transcripts collated were returned to the interviewees for checking and confirmation. The research team then passed the verified transcripts and the original audio recordings to two volunteers from the local sign language association who were certified as sign language interpreters by the Chinese ministry of human resources and social security for secondary proofreading to ensure that the transcripts accurately reproduced the original interview content. Following the methodological guidelines of Marshall, Rossman, and Blanco [25], the research team chose the thematic content analysis method as a means of analyzing the data and summarizing the themes in steps.

## 3. Results

On 23 January 2020, the city of Wuhan, China, the epicenter of the initial COVID-19 outbreak, was placed in a tight lockdown with strict enforcement measures to minimize the movement of people, cancel events and gatherings, and close public places as well as schools and universities to enforce home quarantine [28]. It was not until April 8 of the same year that Wuhan, Hubei Province, announced the lifting of the city-wide lockdown, and daily life gradually returned to normal. This study was conducted six months after the lifting of the blockade, and all 13 respondents experienced the city closure for 76 days due to the specificity of the area they lived in, and had deeper memories of the emergency blockade. After analyzing the in-depth interview data of the 13 interviewees, it is clear that the external communication barriers faced by the older DHH during social isolation were mainly in three areas—information communication barriers, interpersonal communication barriers, and doctor–patient communication barriers.

### 3.1. Information Communication Barriers

Sign language is the first language used by DHH groups for communication, followed by written text. Older DHH people prefer to use natural sign language for communication than the young, and most older DHH people choose to obtain information about the epidemic through mass media channels because they are not proficient in new media technology. For example, they generally choose traditional media such as TV with simultaneous presentation of images and text to receive information. Although China had ratified the United Nations Convention on the Rights of People with Disabilities (UNCRPD) in 2008, and in response to Article 9 of UNCRPD, China promulgated the Regulations on Barrier-free Environment Construction in 2012, which directly contributed to the overall development of “accessibility” practices in traditional media. The regulations specify that television stations established by the municipal governments should create conditions for broadcasting television programs with subtitles and for broadcasting news programs with sign language at least once a week [29]. However, due to the sudden outbreak of the epidemic, the local television station in Wuhan, which was initially located at the epicenter of the epidemic, did not provide sign language interpretation for the epidemic-related broadcasts in a timely manner. If the epidemic information is delivered without the addition of sign language or subtitles on television, older DHH people will not be able to accurately discern and understand the content of the information and may miss receiving important and up-to-date information about the epidemic. More importantly, although there are older hard-of-hearing people who wear hearing aids and use sign language to communicate trying to receive the information about the epidemic through traditional media, medical terminology is inherently difficult to interpret in sign language delivery. What is more, most Chinese DHH people do not understand the CSL gestures on television, and their preference is to use local natural sign language. This invariably raises the threshold for their information reception during the epidemic.

*“Watching TV every day, the TV is always on and I want to know how the epidemic is going now, but you have to know that sometimes the reports on TV are not captioned.”*

*(Interviewee 1)*

*“For me, the most authoritative one is definitely the news broadcast on the local news station, but there is no sign language interpretation, and there is no (sign language interpretation) for the press conference.”*

*(Interviewee 13)*

*“For information about the epidemic, I only watch the sign language broadcasts on TV news, I don’t go online and I don’t look at the information on social media apps on my phone.”*

*(Interviewee 4)*

*“Some news broadcasts have sign language interpretation, but the picture is too small to read.”*

*(Interviewee 4)*

*“90% of the news with sign language I can’t understand, they are with universal sign language ah, it’s different from the one we use.”*

*(Interviewee 3)*

*“You all use WeChat (instant messenger) ah or something to see the epidemic information and emergency notification and so on, but I can’t use my cell phone, I’m talking about the smartphone kind, the operation does not come, it’s too complicated. I don’t go online either.”*

*(Interviewee 9)*

*“The community staff are contacting everyone in the WeChat group, and I am really not skilled in using WeChat, and I can’t look over the too many epidemic notifications in the WeChat group when they come out.”*

*(Interviewee 6)*

*“We deaf people can’t read the news, we don’t know what’s happening outside, and when we react, all the pharmacies have no masks.”*

*(Interviewee 1)*

### 3.2. Interpersonal Communication Barriers

Because of the limitation of social distance, the original social support network maintained by interpersonal communication was cut off by the isolation policy, and older DHH people who were accustomed to offline small circle communication were suddenly plunged into complete social isolation. During the emergency isolation period, older people with hearing loss were unable to engage in basic interpersonal communication, and they were less proficient in social media than younger people, leading to an increased sense of psychological isolation and social alienation. The digital divide and technophobia are particularly pronounced among older DHH individuals because of the strong relationships and circle-group interpersonal communication styles that have solidified over the years, and the accompanying yearly deterioration in reading levels and word processing skills. Even when there were brief opportunities for face-to-face communication, masks and various facial protective gear blocked recognizable communication with each other’s lips and facial expressions in this particular epidemic-proofing situation.

*“There is an old woman named Li in the next building, and her situation is similar to mine. She is deaf, and she goes downstairs to take out the garbage at fixed hours, and sometimes she can stand by the window and meet, but because of the mask, I can’t see her expressions, and I can only judge by gestures, so I can’t communicate at all.”*

*(Interviewee 2)*

*“Originally, my daughter would come to talk to me from time to time, accompany me to go grocery shopping, and she helped me tell others what I wanted, but then the city was closed and she couldn’t come through, so nothing was convenient anymore.”*

*(Interviewee 7)*

*“We have always used local sign language as our mother tongue and we can’t communicate with others in writing, which is different from younger deaf people.”*

*(Interviewee 3)*

*“We have a disability associate in our district who comes once every 2 or 3 days to ask me how I am doing, but he doesn’t know sign language and I don’t know many words, so it’s quite difficult to communicate, and when he comes later, I smile and shake my head to say there is nothing I need.”*

*(Interviewee 12)*

*“There are grid workers in the community who check out the epidemic, sometimes they ask me about my situation by online video, I was living alone, no one helped me communicate with him, it was too difficult to communicate.”*

*(Interviewee 5)*

### 3.3. Doctor–Patient Communication Barriers

Most of the older DHH individuals were suffering from multiple chronic illnesses at the same time, and more than half of the respondents reported that they faced doctor–patient communication problems to varying degrees during the segregation period. Due to isolation policies, family members and sign language interpreters are not allowed to accompany patients in order to minimize infection, which directly contributes to increased communication barriers between these DHH patients and their physicians. In addition to the prevalence of chronic diseases, aging also leads to cognitive decline, and even for general consultations, older deaf and hard of hearing people face more communication difficulties than younger people and take longer to “digest” the medical assistance provided by their doctors. It also exacerbates the barriers to doctor–patient communication for this group during special periods. Specifically, doctor–patient communication barriers for the older DHH population during emergency isolation focused on both medical communication and medical help.

*“I wrote down the questions to ask the doctor and the doctor wrote down the responses, so that the visit indeed take too long……, I chose not to go at the follow-up appointment.”*

*(Interviewee 13)*

*“I hope the hospital will have more sign language interpreters, and I hope the doctors and nurses will be a little more patient with me.”*

*(Interviewee 10)*

*“In February I had acute appendicitis and when everywhere was blocked off, I asked my brother to help me contact the community to take me to the hospital. My brother knew sign language, but because of the quarantine policy he couldn’t accompany me to the hospital, so I had to go by myself. When I got to the hospital, the doctor didn’t know sign language and it took me a long time to talk to the doctor about my illness.”*

*(Interviewee 8)*

*“When I saw the doctor, I sometimes pretended to understand the doctor’s medical advice, he wrote me to read, I knew the words but did not understand what he said the terminology actually meant, and then I did not want to ask the doctor more for fear of trouble.”*

*(Interviewee 11)*

*“In the ward, I tried to call my brother on a WeChat Video and I gestured to him in sign language, and he wrote down what I wanted to say. Later on in the video he showed his text to the medical care that checked the room. So hard to communicate, really.”*

*(Interviewee 8)*

## 4. Discussion

This study is a qualitative study based on in-depth interviews and explores the information communication barriers, interpersonal communication barriers, and doctor–patient communication barriers presented by older DHH individuals in the high-intensity social isolation of an emergency lockdown through thematic content analysis. Through the recovery analysis of the extreme situation in the special period, we comprehensively understand where the external communication barriers of older people with hearing impairment exist during public health emergencies.

China has the largest number of people with hearing disabilities in the world. According to statistics, there are approximately 27.8 million people with hearing disabilities in China, accounting for more than 30% of the total number of people with disabilities in the country [30]. During public health emergencies, older people with hearing impairment have limited access to information and are unable to respond to emergencies such as epidemics in a timely manner due to limited external communication. This makes older DHH people weak in acquiring epidemic-related information on the one hand, and poor in expressing their demands and giving timely feedback on their physical conditions on the other.

During the COVID-19 outbreak, information accessibility continued to be an issue for people with disabilities [2], and this issue was particularly pronounced among the DHH population. Some older DHH individuals have lower information accessibility compared to younger DHH individuals due to a lack of technical resources and/or expertise, which is one of the key reasons for the increased external communication barriers in this group. Older DHH people are particularly behind in accessing information about the epidemic and have a low adoption rate of new media technologies. Some older deaf people never go online and are insulated from almost all news and information. They prefer to read local nature sign language and do not understand text as well as younger DHH people [5]. Although some of the DHH seniors are well educated and have good reading and literacy skills, they automatically choose to avoid textual information that would take much more time to process because they have long been accustomed to use nature sign language with lipreads and maintain strong relational interactions. As a result, older people with hearing loss may be at increased risk of social isolation and loneliness.

With the rapid spread of COVID-19, people with hearing loss have limited access to important public health information because much of it is not available in an accessible format [2]. The World Federation of the Deaf (WFD) and the World Association of Sign Language Interpreters (WASLI) have issued guidelines for accessing public health information, emphasizing the need to ensure that qualified professional sign language interpreters are present on all media channels and platforms and are clearly visible on screen throughout broadcasts [31]. Some of the points in the above interim guidelines have similar corresponding policy provisions in the Regulations on Barrier-free Environment Construction issued by China in earlier years [30].

To some extent, the barriers and challenges for DHH people did receive attention in COVID interventions and policies. On 4 February 2020, the press conference on the prevention and control of the novel coronavirus pneumonia outbreak in Beijing was the first to add sign language interpretation, and the interpreters on the scene drew on foreign experience to wear a homemade mask with a transparent middle part to facilitate DHH people to see facial expressions and understand the sign language content more fully and in place [32]. With the promotion from all over the world, the live broadcast of the State Council’s joint prevention and control mechanism press conference on 10 February, was also equipped with simultaneous CSL interpretation service for the first time, which facilitated the DHH community to obtain the current affairs at the first time and caused great repercussions among the DHH population at home and abroad [33]. On 22 February, a CSL sign language interpreter was present for the first time at the Shanghai COVID-19 outbreak prevention and control conference in response to the difficulties of some DHH people having in accessing information about the outbreak. The first day CSL presenter, Lijun Zhang, stood on the right side of the podium throughout the event and interpreted in real time for all to see. The entire sign language interpretation process was broadcast on the live news screen with one-third screen. It was reported that there were six sign language volunteers for the Shanghai covid-19 epidemic prevention and control press conference series, selected from the Disabled Persons’ Federation and rotating daily. From then on, it has gradually become the norm to have sign language interpreters for news conferences around the epidemic [34]. Admittedly, it will be some time before the commitment to full accessibility of information is fulfilled at the level of the entire social system. There is a need not only to continue to improve the mechanisms for ensuring the rights and interests of persons with disabilities, but also to improve their well-being [35].

During emergency isolation, older DHH people who are unfamiliar with new media technologies and who are not proficient in the use of social media are partially affected in terms of social support due to interpersonal communication barriers. The psychology of isolation brought about by feelings of alienation, coupled with interpersonal communication breakdowns with significant barriers, can easily have an extremely negative impact on health status [36,37]. Interfacing with community emergency workers also presents a large communication barrier to providing services to the DHH population due to their own lack of sign language skills [2]. Evidence from the research literature suggests that in the past, many people with hearing loss were virtually disconnected from local community services and rarely reached out for help because of communication difficulties. The study also reflects a core issue regarding “empty nod”, whereby many deaf people often answer “yes” to questions they do not understand during doctor–patient communication in order to avoid appearing ignorant [38,39]. This phenomenon was particularly acute during the COVID-19 outbreak. For example, the “empty nod” behavior reported by respondents in this study is an avoidance type of dealing with the fear of facing communication barriers.

In addition, there is a deeper reason for the DHH population’s external communication dilemma during the social isolation period, and that is that the official CSL used in public is not fully compatible with the cultural language of the deaf community. The cultural language of the deaf community in China is local natural sign language, similar to the situation in most countries, which does not translate easily into syntactic and grammatical Chinese. The CSL, which was launched in 2018, is precisely based on a Chinese grammatical system to create the basic word list, which made the initial rollout of CSL nationwide face considerable difficulties. These difficulties arise not only from the ideographic distinction between natural and grammatical sign languages, but also from the differences in dialectal expressions between different regions. The prerequisite for safeguarding the language communication rights of special populations in the normalized phase of epidemic prevention and control is to provide a good ecological environment. On 20 October 2020, the China Disabled Persons’ Federation, together with the Central Propaganda Department, the Ministry of Civil Affairs, and other departments and units, organized the development of the Guidelines for Social Support Services for Protection of Persons with Disabilities during Major Infectious Disease Epidemics, with the aim of standardizing and leading, publicizing and guiding, and socially advocating, through standards promote the standardization, standardization and normalization of public services related to the protection of persons with disabilities under epidemics, help persons with disabilities overcome the special difficulties and special risks brought by epidemics, and guarantee equal rights of persons with disabilities to enjoy public health security [40]. However, the above-mentioned measures depend on a long and continuous promotion to be fulfilled.

The global COVID-19 Disability Rights Monitor (COVID-19 DRM) report identifies the impact of the pandemic on people with disabilities as “catastrophic” and calls for “urgent action” to safeguard the rights of people with disabilities [41]. “The COVID-19 outbreak highlights existing inequalities and marginalization in the disability community, with older DHH people becoming particularly vulnerable during the pandemic due to limited communication options. Studies have confirmed that older adults with hearing disabilities are vulnerable to the COVID-19 pandemic and face systemic challenges as medical services and medications become scarce [42]. Compared to normal days, the COVID-19 outbreak presents a more physician-patient communication state, and older adults with hearing loss are unable to access medical resources through smoother external communication pathways. DHH individuals with multiple chronic conditions are reluctant to seek care because of the long wait times in health care facilities. Of course, telemedicine can be a means of enhancing the patient experience and expanding access to health care resources. For example, on the evening of 11 February 2020, Chen Xin, a Post-00s sign language interpreter volunteer in Nanjing, saw in a sign language exchange group that someone had posted information about Wuhan Vulcan Mountain Hospital’s recruitment for sign language interpreters, “as a subconscious reaction, and immediately signed up after seeing it.” He learned that Wuhan Vulcan Mountain Hospital had received a 75-year-old older female DHH patient, and that it was very inconvenient for the doctor to communicate with her about her condition, so a sign language interpreter was urgently needed. That night, Chen Xin made an appointment with the medical staff to help the patient interpret by video communication the next day. Subsequently, he and the patient made several connections to exchange relevant conditions and help the doctor–patient communication. However, point-to-point communication support on a case-by-case basis is not enough. The diffusion and dissemination of digital technology for telemedicine that supports intelligent sign language interpretation should be on the agenda. This digital technology can compensate for communication barriers that hinder access to public services. It is worth thinking about how the tens of millions of people with disabilities in the prevention and control emergency system can actually be optimized through more inclusive policy practices for doctor–patient communication.

## 5. Limitations

There are potential limitations to this study. Firstly, this study is a retrospective interview study and may suffer from some memory biases. Secondly, this study is a small sample and some of the results are not representative in the full sense of the word. Finally, this study focused on Wuhan, China, the epicenter of the initial outbreak with regard to the special period of the emergency blockade, new issues may arise in other contexts, for example, some of the target groups with lower education and rural residence may face a completely heterogeneous difficult situation.

## 6. Conclusions

This study focuses on DHH seniors’ experiences of external communication barriers in the initial phase of the epidemic, how they dealt with those experiences, and their perspectives toward their external communication barriers, and uncovered the multifaceted phenomena with regard to the external communication coordination mechanisms for DHH people in the epidemic. The participants of this study gave feedback that hearing impairment and poor physical health posed difficulties in terms of information access, i.e., delayed access to information because of the cut-off of the social support system and emergency protection materials. The DHH people are further limited in communication methods and channels than usual, and they have additional difficulties in receiving accurate information due to their weak risk-resistance and self-help ability, which lead them to misperception of the epidemic and to greater risk of infection. In the emergency management of the pandemic, it is of great importance to strengthen and standardize the social support services for the disabled groups, and to protect the lives, health and interests of the disabled to the maximum extent.

Neither the blind belief that a transparent mask is a one-size-fits-all solution to the communication problems of all older adults with hearing loss, nor the simple belief that external communication difficulties can be better improved through online remote services, is a reasonable solution for the creation of a long-term accessible information environment. Perhaps an alternative approach to consider practical and effective countermeasures to maximize the ability of COVID-19 healthcare providers to provide unhindered doctor–patient communication to older DHH people is a more feasible improvement option. For example, from creating easy-to-identify sign language “keyword cards” for common medical terms, which could be implemented on a large-scale during telemedicine. Another option would be adding a basic online sign language interpretation service at medical facilities. Further, when an outbreak news conference is broadcast live to convey emergency information, a sign language interpreter could be presented on a half screen instead of a fifth of the screen in the lower left corner. In conclusion, to protect vulnerable groups of older DHH people in the COVID-19 pandemic and situations alike, extra efforts are urgently called for from the local to national level to improve the recognition of the vulnerability of this special population and to provide more inclusive communication methods and mechanisms for people with disabilities.

## Figures and Tables

**Table 1:** Basic Characteristics of Respondents.

ID	Sex	Age	Deaf/ Hard-of-Hearing	Education	Residence	Urban Center (Yes/No)	Treatment Experience	Duration of Medical Treatment	Understanding of Medical Advice
1	Male	63	Deaf	Undergraduate	Qingshan	Y	Consultations for chronic diseases	/	/
2	Male	67	Hard-of-hearing	Undergraduate	Jianghan	Y	Cured by self-medication at home	/	/
3	Female	76	Deaf	Elementary school	Hanyang	Y	None	/	/
4	Male	69	Hard-of-hearing	Undergraduate	Hanyang	Y	Consultations for chronic diseases	2 h	Mostly understood
5	Male	66	Hard-of-hearing	Middle school	Qiaokou	Y	None	/	/
6	Female	65	Deaf	Elementary school	Jiangan	Y	None	/	/
7	Female	71	Deaf	Elementary school	Jianghan	Y	None	/	/
8	Male	72	Deaf	High school	Hannan	N	Hospitalized for acute illness	1 h	Mostly understood
9	Female	70	Deaf	High school	Wuchang	Y	Consultations for chronic diseases	1 h	Fully understood
10	Female	61	Deaf	Postgraduate	Dongxihu	N	Consultations for chronic diseases	2 h	Not understood at all
11	Male	62	Hard-of-hearing	Junior college	Qiaokou	Y	Consultations for chronic diseases	2 h	Partially understood
12	Male	66	Hard-of-hearing	High school	Wuchang	Y	None	/	/
13	Male	62	Hard-of-hearing	Postgraduate	Hanyang	Y	Consultations for chronic diseases	2 h	Mostly understood

## Data Availability

The data provided in this study are available upon request from the corresponding authors. Data in the administrative Excel file are not publicly available because the information may compromise the privacy of study participants.
